# The Role of Physical Activity and Mortality in Hemodialysis Patients: A Review

**DOI:** 10.3389/fpubh.2022.818921

**Published:** 2022-02-17

**Authors:** Fan Zhang, Hui Wang, Weiqiong Wang, Huachun Zhang

**Affiliations:** ^1^Department of Nephrology, Longhua Hospital Shanghai University of Traditional Chinese Medicine, Shanghai, China; ^2^Department of Anorectal, Longhua Hospital Shanghai University of Traditional Chinese Medicine, Shanghai, China; ^3^Blood Purification Centre, Longhua Hospital Shanghai University of Traditional Chinese Medicine, Shanghai, China; ^4^Department of Nursing, Longhua Hospital Shanghai University of Traditional Chinese Medicine, Shanghai, China

**Keywords:** physical activity, hemodialysis, mortality, strategy, review

## Abstract

Available data indicated that physical activity was related to improved outcomes in hemodialysis patients. Multiple observational studies involving different cohorts have reported that increased physical activity level was associated with decreased mortality among hemodialysis patients. Therefore, promoting physical activity has become an increasingly critical and promising approach to improving cardiovascular health and clinical outcomes in hemodialysis patients. This review summarizes the published articles regarding physical activity and hemodialysis patients, focusing on mortality and strategy to promote physical activity.

## Introduction

End-stage renal disease (ESRD) is a global health problem characterized by high morbidity and mortality (1). About 600,000+ ESRD patients receive renal replacement therapy, and hemodialysis (HD) accounts for 91.94% in China (2). In recent years, patients with ESRD have increased life expectancies because of advances in HD (3). Despite this, the risk of premature death in HD patients is 20 times higher than in the general population due to cardiovascular disease in more than 50% of dialysis patients (4). It causes a burden to the afflicted individuals and their families and the healthcare system (5).

Physical activity refers to any bodily movement produced by skeletal muscles that require energy expenditure (6). Evidence showed that being physically active is vital for maintaining health and preventing the exacerbation of diseases in all populations (7), including patients with ESRD treated by hemodialysis (8). Even light physical activity has been inversely associated with the risk of death among patients with renal disease (9). Importantly, regular physical activity is beneficial across all stages of renal disease, improving physical fitness, muscular strength, and health-related quality of life (10).

Nevertheless, HD patients engage in less physical activity than healthy age-matched controls (11). Meanwhile, inactivity worsens over time and with disease severity (12). Prolonged time spent sedentary was associated with an increased risk of all-cause mortality in HD patients (13, 14). Accordingly, there are growing voices to integrate physical activity into routine care among HD patients (15).

This review summarized the available data regarding the associations between physical activity and mortality among HD patients. On the basis, we showed the results of recent randomized controlled trials (RCTs) on modifying physical activity in HD patients and concluded some recommendations for future research.

## Physical Activity and Mortality: Data From Cohort Studies

**Figure 1:**
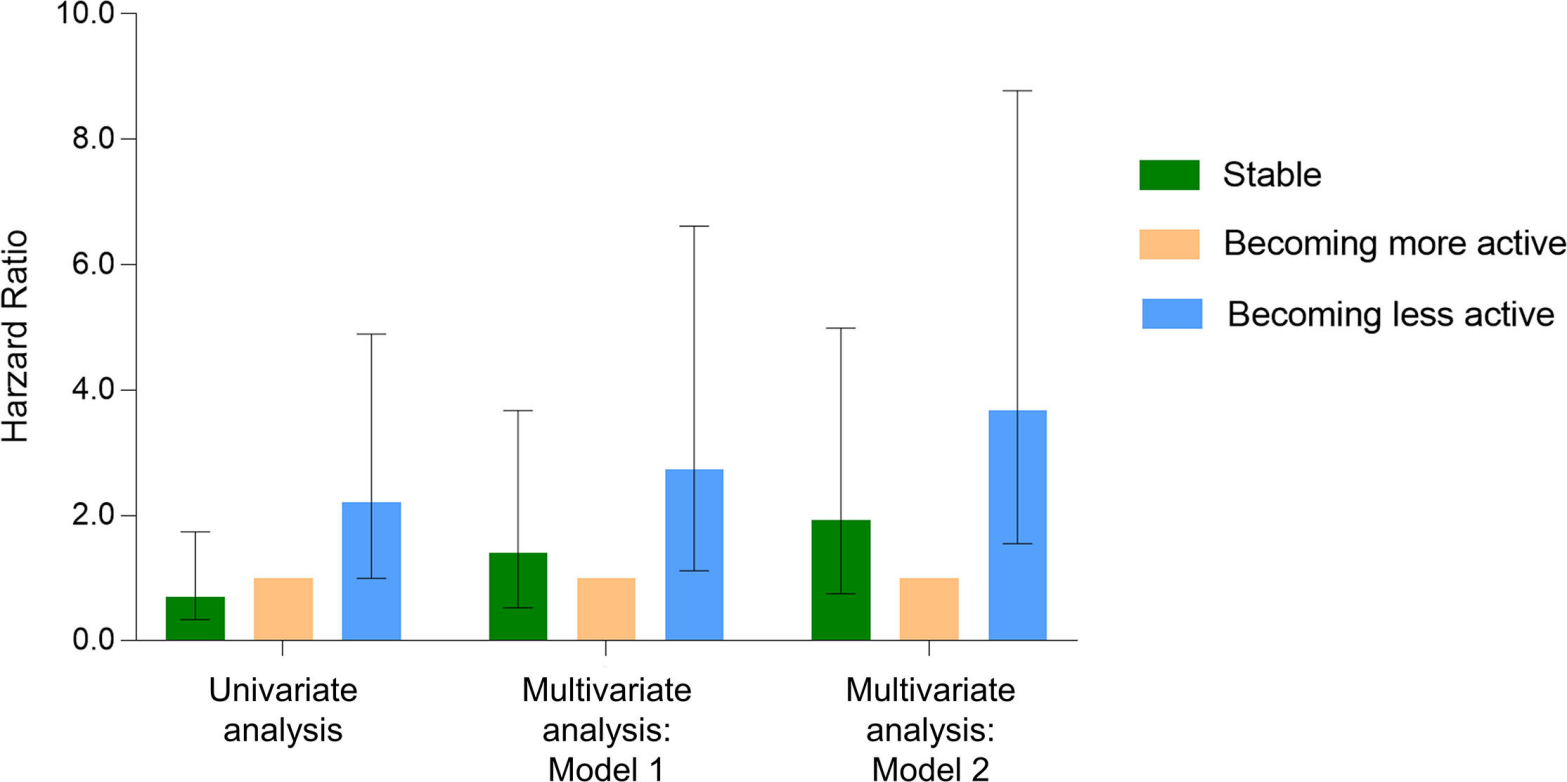
Cox proportional hazards model for all-cause mortality in hemodialysis patients. Change in physical activity concerning the risk of all-cause mortality among 192 HD patients with a 7-year follow-up. **Crude model:** Compared to the *becoming more active group*, the hazard ratios (HR) for all-cause mortality in the *becoming less active group* and the *stable group* were 2.21 (95% confidence interval (CI): 1.00–4.89; *P* = 0.05) and 0.70 (95% CI: 0.34–1.74; *P* = 0.54), respectively. **Model 1:** Compared to the *becoming more active group*, the HR for all-cause mortality in the *becoming less active group* and the *stable group* were 2.73 (95% CI: 1.12–6.62; *P* = 0.03) and 1.41 (95% CI: 0.53–3.67; *P* = 0.49), respectively. Adjusted for age, sex, time on hemodialysis, body mass index, primary kidney disease, atherosclerotic heart disease, congestive heart failure, cerebrovascular accident/transient ischemic attack, diabetes, serum albumin, and baseline physical activity. **Model 2:** Compared to the *becoming more active group*, the HR for all-cause mortality in the *becoming less active group* and the *stable group* were 3.68 (95% CI, 1.55–8.78; *P* < 0.01) and 1.93 (95% CI: 0.75–4.99; *P* = 0.17), respectively. It was adjusted for the comorbidity index effect, which consisted of a cause of end-stage renal disease and 11 comorbidities. Data adapted.

Regular physical activity is associated with a reduced risk of mortality caused by cardiovascular disease, diabetes, obesity, and cancer (16, 17). Substantial prospective observational studies of HD patients support the same conclusion (Table 1). In a recent meta-analysis of nine cohort studies, greatest vs. lowest physical activity levels was associated with a 14% lower risk of all-cause mortality (hazard ratio (HR) = 0.86, 95% confidence interval (*CI*), 0.83 to 0.89; *P* = 0.003) in patients with end-stage renal disease (23). Data from the Dialysis Outcomes and Practice Patterns Study (DOPPS) revealed that mortality risk was lower among patients who engage in regular physical activity (HR = 0.73, 95% *CI*, 0.69 to 0.78; *P* < 0.001) (24). However, data are limited regarding whether changing physical activity among HD patients is associated with improved mortality. One retrospective analysis in a study of 192 HD patients reported that those who remained stable PA or becoming less active had a 0.93 (95% *CI*, 0.75 to 4.99; *P* = 0.17) and 2.68 (95% *CI*, 1.55 to 8.78; *P* < 0.01) higher risk of all-cause mortality compared to patients becoming more active, independent of the baseline physical activity and patient's characteristics (Figure 1) (25). Taken together, these data support the conclusion that higher levels of physical activity are associated with lower mortality. However, the mechanisms underlying this effect are unknown and still need to be investigated.

**Table 1:** Studies of the association of PA with mortality in HD patients.

**References**	**Sample**	**Method**	**Follow-up (years)**	**Finding**
Hishii et al. (13)	71	Sedentary behavior was measured using an accelerometer.	Mean 3.0 ± 1.7	HR 2.83 (95% *CI*, 1.11 to 7.32; *P* = 0.028) for the total days, 2.98 (95% *CI*, 1.21–8.03, *P* = 0.016) for the HD days, 2.79 (95% *CI*: 1.13–7.49, *P* = 0.024) for the non-HD days. Reference group: short-sedentary behavior group (patients with a relative value (%) of daily wearing tri-accelerometer time below the median).
Matsuzawa et al. (18)	282	Physical activity was measured by an accelerometer.	Median 4.7 (IQR 2.4–7.0)	HR 2.37 (95% *CI* 1.22 to 4.60, *P* = 0.01) for daily step counts < 4,000. Reference group: ≥ 4,000 steps
Lopes et al. (19)	5,763	Physical activity was measured using the Rapid Assessment of Physical Activity (RAPA).	Median 1.6 (IQR 0.9–2.5)	HR 0.89 (95% *CI*, 0.72 to 1.10) for infrequently active, 0.84 (95% *CI*, 0.67 to 1.05) for sometimes active, 0.81 (95% *CI*, 0.68 to 0.96) for often active, and 0.60 (95% *CI*, 0.47 to 0.77) for very active group (*P* for trend < 0.001). Reference group: inactive (each question of RAPA has a “Yes” or “No” option. The score ranges from 1 to 7; a score of 6–7 points is considered “very active,” 4–5 points as “often active,” and ≤ 3 points is defined as “inactive” to “sometime active”).
Zhang et al. (20)	317	Physical activity was measured by using the Human Activity Profile and the Stanford 7-day Physical Activity Recall Questionnaire (PAR).	3.0	Mortality risk decreases by 0.44 for each point increase in the maximal activity score, decreasing by 0.69 for each increase in light physical activity time and by 0.66 for each point increase in the PAR.
Matsuzawa et al. (21)	202	Physical activity was measured by an accelerometer.	Mean 3.75 (range 0.2–7.0)	HR 0.78 (95% *CI*, 0.66 to 0.92; *P* = 0.002) per 10 min/day increase physical activity.
O'Hare et al. (22)	2,264	A single question: How often do you exercise (do physical activity during your leisure time)?	Median 1.0 (no IQR)	HR 1.62 (95 *CI* 1.16 to 2.27). Reference group: non-sedentary (responding to the questionnaire as “almost never or never” was defined as sedentary).

*HR, hazard ratio; IQR, interquartile range; CI, confidence interval*.

## Strategy to Promote Physical Activity: Data From Randomized Controlled Trials

Given the insufficient levels of physical activity present among HD patients and the links between sedentary lifestyle and the higher mortality, interventions are needed that can promote HD patient's physical activity. Nine RCTs have evaluated the effects of different strategies on physical activity and have had inconsistent results (Table 2). As a whole, these interventions include (a) pedometer-based intervention based on goal-setting theory; (b) physical activity increase based on exercise interventions to improve physical function; (c) changing the dialysis model.

**Table 2:** Summary of RCTs for promoting physical activity in HD patients.

**References**	**Physical activity measure**	**Intervention**	**Findings**
Sheshadri et al. (26)	Physical activity was measured as a daily step count using a pedometer.	Providing pedometers in conjunction with weekly semi-scripted counseling sessions, patients were also set a goal of increasing their step count by 10% compared to the previous week.	After 3 months, patients in the intervention increased their average daily steps by 2,256 (95% *CI*, 978 to 3,537) more than the controls (*P* < 0.001).
Ortega et al. (27)	Physical activity measured by Human Activity Profile.	Progressive exercise intervention at home or during dialysis over 16 weeks	Both interventions were equally effective at increasing physical activity levels (intradialytic group increases from 62.4 ± 16.6 to 67.3 ± 15.6; home-based group increases from 51.1 ± 18.1 to 54.3 ± 19.3) among the participants after 16 weeks with a significant time effect (*P* = 0.012).
Cho et al. (28)	Physical activity measured by a triaxial accelerometer	A 12-week intradialytic exercise program (3 times/week).	Patients in the aerobic exercise (1.02 ± 0.03 vs. 1.04 ± 0.04, *P* = 0.04) and combine exercise (1.06 ± 0.05 vs. 1.09 ± 0.08, *P* = 0.01) group increased their metabolic equivalent at 12 weeks.
Assawasaksakul et al. (29)	Physical activity measured as daily step count using a wrist-worn triaxial accelerometer	A 6-month intradialytic cycle ergometer for 60 min.	The physical activity in the exercise group was significantly increased from 5,613 to 8,725.1 steps/day in the sixth month (*P* = 0.046).
Martins et al. (30)	Physical activity was measured using a triaxial accelerometer	A 12-weeks moderate-intensity intradialytic resistance training	After 12 weeks, patients in the exercise group increased their average daily steps by 1,457.8 (95% *CI*,−232.6–3,148.2) than the controls (*P* = 0.22).
Dong et al. (31)	Physical activity was measured by the International Physical Activity Questionnaire.	A 12-week progressive intradialytic resistance exercise with high or moderate intensity (3 times/week).	After the 12-week intervention, the differences were statistically significant in physical activity level between groups (*P* < 0.05).
Koh et al. (32)	Physical activity was measured by the International Physical Activity Questionnaire.	Intradialytic exercise 3 times/week for 6 months on a cycle ergometer and home-based exercise followed a walking program.	Self-reported physical activity increased in the intradialytic exercise group (*P* = 0.03) but not in the home-based exercise group (*P* = 0.3).
Pecoits et al. (33)	Physical activity measured as daily step count using an accelerometer	Patients receive high-volume online hemodiafiltration (HDF) or HD	Patients received HDF was +538 (95% *CI*−330 to 1,407) steps/24 h compared with HD, but no statistically significant (*P* = 0.262).
Bohm et al. (34)	Physical activity was measured using a biaxial accelerometer	Intradialytic exercise 3 times/week for 6 months on an ergometer and home-based walking program using a pedometer.	There was no statistically significant change in the amount of physical activity at any intensity level over time in either group (*P* > 0.05).

*CI, confidence interval*.

While some studies have shown positive effects, another portion of the non-statistically significant results warrant further thought: (a) since self-reported physical activity is unreliable, wearable devices, represented by pedometers and accelerometers, were gradually being used for physical activity assessment in HD patients (35). However, pedometers may be insensitive to detect activity in patients who walk slowly (36). Tri-axis accelerometer, more likely to detect dynamic changes in physical activity (37); (b) the choice of physical activity outcome assessment may determine the success or failure of different measures in promoting physical activity effects. Most RCTs have focused on the impact of interventions on daily step count. It has been suggested that increases in daily steps are not necessarily accompanied by increases in metabolic equivalents or time spent in moderate to vigorous-intensity physical activity (38); (c) given that minor changes in physical activity can elicit health benefits, it may be necessary to transition our thinking from “promoting physical activity” to “active living” (39). This concept includes leisure and domestic activities and transportation (walking and bicycling), although these activities are not necessarily detected by the available measurement tools.

## Future Directions

HD patients were generally inactive, and this sedentary nature was detrimental to both quality and quantity of life. Therefore, increased efforts to understand better the determinants of physical activity and practical strategies to improve this variable should be a principal goal in promoting a physically active lifestyle among HD patients. Future research should focus first on the best tools for measuring physical activity for HD patients in clinical practice. The ability to accurately measure physical activity is not optimal in HD patients that are commonly slow-moving (40). The best available device is an accelerometer, yet few have been robustly validated for use in the public. Concerning the few validated accelerometers, undercounting has been shown compared to pedometers (41). There is a significant need to establish a proper and affordable way to objectively measure physical activity in slower-moving populations, particularly for lighter activity levels. This likely resides in accelerometry and the development of more advanced algorithms to account for more finite patterns in the accelerations on two to three axes in various devices.

Secondly consider strategies that best promote physical activity in HD patients, including increasing activity over time. Healthcare system-level is a common barrier to physical activity participation for HD patients (42). For example, dialysis care providers may lack exercise-related knowledge, access to exercise education resources, or time to provide education. Indeed, HD patients are at high risk for cardiovascular disease, and staff support for exercise programme is critical to its success (43). This role can be performed by nurses who have attended specially trained or other staff with a specific interest in exercise. They should understand the benefits of exercise for HD patients, know how to get them started on an exercise programme, and motivate them to continue exercising. Other members of the multidisciplinary team, such as kinesiologists/physiologists, may be a critical factor in the sustainability of physical activity management (44). A review by Bennett et al. (45) reported that the most common factor affecting the sustainability of hemodialysis exercise programmes was the availability of dedicated exercise professionals. Future research should consider the specific value of sports medicine in managing physical activity in hemodialysis patients. And then address the following direction:

1. Potential Disease-Modifying Effects of Increased Physical Activity Levels in HD Patients.
2. Determinants of Physical Function Improvement Among HD Patients Are Helpful to Increase the Level of Physical Activity.
3. The Association of Different Intensities (Light/Moderate/ Vigorous Intensity) Physical Activity With Prognosis (Including Health Status and Mortality) in HD Patients.
4. Conduct High-Quality RCTs to Assess Whether Increases in Physical Activity Resulting From the Intervention Lead to Improved Health Outcomes.
5. Tailor Physical Activity to the Individual HD Patient, Taking Into Account Exercise Capacity, Illness and/or Disability, Family and Community Environment, and Behavioral and Cultural Factors.

## Summary

Healthcare workers should educate HD patients that physical activity is practical, safe, and feasible for most individuals. HD patients can benefit from even low-intensity physical activity to change health outcomes. During recent years, several clinical guidelines have published to aid physical activity management for HD patients (46, 47). Further, to develop strategies to increase physical activity and improve health outcomes in chronic kidney disease, the Global Renal Exercise Network (GREX) provides a compilation of available resources to serve patients with all stages of kidney disease (https://grexercise.kch.illinois.edu/). Considering the substantial cost associated with HD treatment and the somewhat theoretical benefit of physical activity, we should focus on keeping HD patients participating in physical activity. Therefore, promoting physical activity should be integrated into lifestyle management for HD patients.

## Author Contributions

HW and FZ: conceptualization. WW: writing-original draft preparation. HZ: writing-review and editing. All authors have read and agreed to the published version of the manuscript.

## Funding

The study was supported from Longhua Hospital Shanghai University of Traditional Chinese Medicine (Grant Number: Y21026).

## Conflict of Interest

The authors declare that the research was conducted in the absence of any commercial or financial relationships that could be construed as a potential conflict of interest.

## Publisher's Note

All claims expressed in this article are solely those of the authors and do not necessarily represent those of their affiliated organizations, or those of the publisher, the editors and the reviewers. Any product that may be evaluated in this article, or claim that may be made by its manufacturer, is not guaranteed or endorsed by the publisher.
